# Association of Handgrip Strength with Diabetes, Hypertension, and Comorbidities in a Korean Population: A Large-Scale Cross-Sectional Study

**DOI:** 10.3390/jcm14082801

**Published:** 2025-04-18

**Authors:** Bum Ju Lee

**Affiliations:** Digital Health Research Division, Korea Institute of Oriental Medicine, Daejeon 34054, Republic of Korea; bjlee@kiom.re.kr; Tel.: +82-42-868-9593

**Keywords:** anthropometry, comorbidity, diabetes, handgrip strength, hypertension

## Abstract

**Background:** Handgrip strength (HGS) is strongly associated with hypertension (HTN), diabetes mellitus (DM), and HTN and DM comorbidity (HDC). However, no studies have simultaneously examined anthropometric and absolute/relative HGS indices among HTN, DM, and HDC patients. The objective of this study was to examine the associations of anthropometric and HGS indices with HTN, DM, and HDC. **Methods:** For this large-scale cross-sectional study, we used a dataset from the Korea National Health and Nutrition Examination Survey. The study population included 15,343 participants aged 50 years and older. Complex sample binary logistic regression was used to examine the associations of each disease with the anthropometric and HGS indices in crude and adjusted models. **Results:** The prevalence of HTN, DM, and HDC in the study population was 35.1%, 8.6%, and 14.4% in men and 34.7%, 5%, and 11.8% in women, respectively. In men, the indices with the strongest associations with HTN, DM, and HDC were body mass index, waist circumference, and waist-to-height ratio. Among women, body mass index and waist-to-height ratio had the strongest associations with HTN. Waist circumference and waist-to-height ratio had the strongest associations with DM. Waist-to-height ratio had the strongest associations with HDC. Additionally, the relative HGS indices were more strongly associated with these diseases than the absolute HGS indices. **Conclusions:** HGS indices were associated with HTN, DM, and HDC, but compared with the absolute HGS index and relative HGS indices, anthropometric indices were more strongly associated with these diseases in the Korean population.

## 1. Introduction

Hypertension (HTN) and diabetes mellitus (DM) are the most important risk factors for heart disease, stroke, cardiovascular disease and morbidity in the US [1,2,3,4,5,6]. While the prevalence of HTN has decreased in high-income countries such as the US, the prevalence of diabetes is increasing worldwide [1,2,3,4,5,6]. Additionally, the prevalence of both HTN and DM is greater in low- and middle-income countries than in high-income countries [2,5]. Although HTN and DM are caused by different biological and pathophysiological mechanisms [6], these diseases share common risk factors, such as obesity, low physical exercise, high alcohol consumption, high salt intake, and low handgrip strength (HGS) [6,7,8,9,10]. HTN and DM are well-known complications of each other, and the risk of cardiovascular events in patients with HTN and DM coexistence (HDC) is 5 times greater than that in healthy individuals [6,7]. The mechanism of HDC may be partially explained by insulin resistance [3,11]. Many patients with HTN have hyperinsulinemia, and insulin resistance induces HTN through increased sodium reabsorption and stimulation of the sympathetic nervous system [11,12].

Recently, HGS has been associated with DM, HTN, and various chronic diseases, such as cardiovascular disease, mental health, cancer, and kidney disease [13,14]. Indeed, many studies have suggested that HGS is strongly associated with HTN and DM [8,14,15,16,17,18,19,20,21] and that subjects with HTN or DM have lower HGS than those without HTN [8,18,19,21,22] or DM [8,14,15,16,17,19,20,23,24,25,26,27]. For example, in a study on the association of HGS with DM and HTN based on data from the National Health and Nutrition Examination Survey 2011–2012 in the US, HGS was significantly lower in individuals with HTN and DM compared with individuals without HTN and DM [8]. Most recently, associations between HGS and various chronic diseases, including cardiovascular disease, HTN, DM, and mental health, have been reported in a narrative review of many studies [14]. Furthermore, some studies have suggested that both absolute HGS and relative HGS (HGS divided by body mass index, BMI) were significantly associated with HTN and DM and that relative HGS was more powerful for screening for HTN compared with absolute HGS [18,28]. Therefore, these studies suggest that HGS can be considered a new predictor for HTN and DM in clinical practice. Although numerous studies have been conducted to reveal the associations of HGS with HTN and DM, there are no studies examining anthropometric and absolute/relative HGS indices in individuals with HTN, DM, and comorbidities to identify better indicators of these diseases.

Therefore, the objective of this study was to examine the associations of HGS and anthropometric indices with HTN, DM, and HDC in the Korean population. We believe that examining HGS and anthropometry as risk factors for HDC is important because patients with HDC have a much greater risk of cardiovascular events than healthy subjects [3,7]. To our knowledge, this is the first study to examine HTN, DM, and HDC with anthropometry and HGS indices.

## 2. Materials and Methods

### 2.1. Study Design and Target Population

This large-scale cross-sectional study was based on the Korea National Health and Nutrition Examination Survey performed by the Korea Disease Control and Prevention Agency in the Republic of Korea. The target population of the Korea National Health and Nutrition Examination Survey was nationally representative and included noninstitutionalized civilians older than 1 year of age. All participants were selected based on stratification, clustering, and weighting sampling according to age, residential area, and sex to represent the entire Korean population. Therefore, the Korea National Health and Nutrition Examination Survey dataset includes nationally representative and credible statistics on socioeconomic and sociodemographic information, health-related behaviors, blood parameters, clinical profiles, physical examinations, and nutritional components in the South Korean population [9,10,29,30,31,32]. This health survey dataset was collected via interviews with well-trained staff and experts to reduce respondent recall bias. Additionally, health examinations were carried out by direct laboratory analyses and measurements. The survey was performed with the approval of the Institutional Review Board (IRB) of the Korea Disease Control and Prevention Agency (IRB no. 2018-01-03-P-A, 2018-01-03-C-A, and 2013-12EXP-03-5C) [29,30,31]. We also received approval from the IRB of the Korea Institute of Oriental Medicine (IRB No. I-2501/001-001) for the use of the Korea National Health and Nutrition Examination Survey data.

### 2.2. Study Population

**Figure 1:**
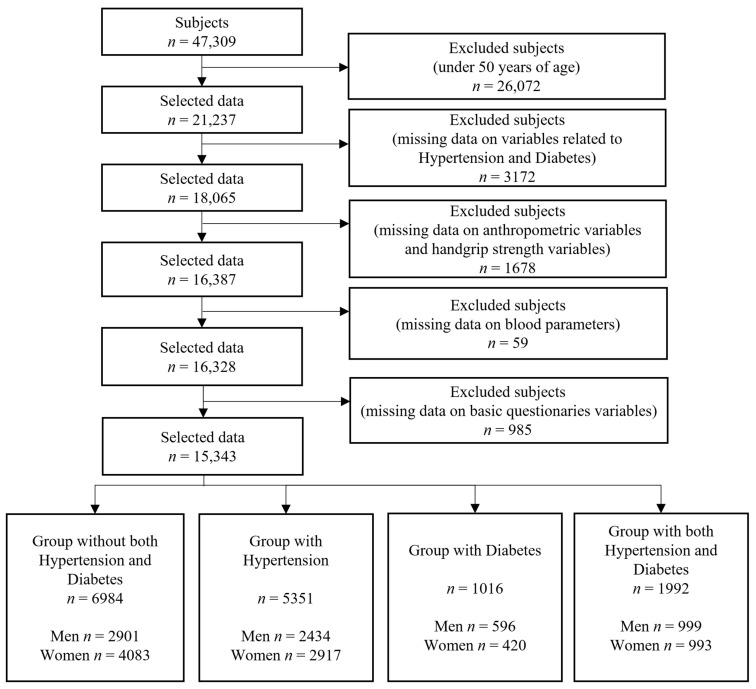
Sample selection process.

In the Korea National Health and Nutrition Examination Survey, HGS measurements began in 2014. Therefore, HGS and anthropometric data from 2014 to 2019 were used for this study; data for a total of 47,309 subjects were evaluated. For internal validity, this dataset was collected via interviews with experts to reduce respondent recall bias. Many covariates used in previous studies were used in our adjusted models. Also, we selected adult subjects aged 50 years and older because the prevalence of HTN and DM comorbidity was extremely low in people under 50 years of age. We excluded participants with missing values for important variables, such as target disease-related variables, HGS measurements, anthropometry, basic sociodemographic characteristics, and blood profiles. A total of 15,343 participants (6930 men and 8413 women) were included in the statistical analysis. The specific inclusion and exclusion criteria and procedures are shown in Figure 1. This study was performed in accordance with the principles of the Helsinki Declaration, and written informed consent was obtained from all participants. All procedures and methods were performed in accordance with the guidelines of the Korea Disease Control and Prevention Agency [29,30,31].

### 2.3. Definitions of Hypertension, Diabetes, and Comorbidities

DM was defined as doctor-diagnosed diabetes, intake of antidiabetic medication, insulin injection, or fasting plasma glucose ≥ 126 mg/dL. HTN was defined as the intake of antihypertensive medication, diastolic blood pressure (DBP) ≥ 90 mmHg, or systolic blood pressure (SBP) ≥ 140 mmHg. The HTN group comprised patients with HTN but not DM, and the DM group comprised patients with DM but not HTN. HDC was defined as having both DM and HTN. The non-HDC group comprised patients who did not have HTN or DM. The definitions of HTN and DM refer to the guidelines from the Korea Centers for Disease Control and Prevention [29,30,31].

### 2.4. Covariates

Based on our literature review, we used several relevant covariates for adjustment. The crude model was not adjusted for any covariates. The covariate included in Model 1 was age only [9,18,28,33,34,35,36]. The covariates used in Model 2 were age [9,18,28,33,34,35,36], residential area (dichotomized into urban and rural areas) [9,18,37], household income (quartiles of income) [34], employment status (yes or no) [9,18,36], education level (categorized into elementary school or below, middle school, high school, or university or above) [9,18,28,34,36,38], alcohol consumption (yes or no) [9,18,28,33,35,36,38], smoking status (categorized into everyday, former, or never) [9,18,20,28,33,34,35,36,38], total cholesterol [34,39], HDL cholesterol [34,39], and triglycerides [33,39]. Additionally, we added one covariate for menopause for women (yes or no) [9,38].

### 2.5. Measurements

All anthropometric and HGS indices of the subjects were measured by well-trained experts or staff according to standardized protocols related to the measurements. For measurements of anthropometric indices, body height and weight were assessed by automatic equipment in units of 0.1 cm using a Seca 225 portable stadiometer (Seca, Hamburg, Germany) and 0.1 kg using an electronic scale (GL-6000-20; G-Tech International, Seoul, Republic of Korea). WC was assessed by tape measurement at 0.1 cm using a SECA 200 (Seca, Hamburg, Germany). WHtR was estimated as WC divided by height. SBP and DBP were measured three times by a standard mercury sphygmomanometer (Baumanometer Wall Unit 33 (0850), Copiague, NY, USA), and we used the mean of the second and third measurements. Among the HGS measurements, dominant HGS and nondominant HGS were measured by a digital grip strength dynamometer (T.K.K 5401, Japan; Takei Scientific Instruments Co., Ltd., Tokyo, Japan). HGS was measured three times, and the average of the HGS values was used for this study. Specifically, the absolute HGS indices were the maximum handgrip strength of the dominant hand (HGS-DH) and the maximum handgrip strength of the nondominant hand (HGS-non-DH). Relative HGS indices were obtained as HGS-DH and HGS-non-DH divided by BMI, WC, and WHtR. Therefore, the relative HGS indices were HGS-DH/BMI, HGS-DH/WC, HGS-DH/WHtR, HGS-non-DH/BMI, HGS-non-DH/WC, and HGS-non-DH/WHtR. Details of the HGS and anthropometric measurements were provided in previous work [10,40,41,42,43,44].

### 2.6. Statistical Analysis

All of the statistical analyses were performed using the software IBM SPSS 28 (IBM SPSS, Inc., Chicago, IL, USA). For analysis of sex differences, *t* tests based on a general linear model and Rao–Scott chi-square tests were used for continuous variables and categorical variables, respectively. Associations of each disease with anthropometric and HGS indices were obtained by complex sample binary logistic regression after applying standardization. Due to the complex survey sample design, stratification, clustering, and weighting were applied to the Korea National Health and Nutrition Examination Survey dataset to represent the entire Korean population. Details of the complex survey sample design of the Korea National Health and Nutrition Examination Survey are described elsewhere [10,41,42]. We considered three models according to unadjusted and adjusted covariates for both men and women, as described in the Covariates subsection. Before analyzing the data, we tested the variance inflation factor to avoid potential multicollinearity between variables (variance inflation factor < 5). Furthermore, we examined the linearity between the logit of independent and dependent variables using the Box–Tidwell test. Complex sample binary logistic regression was used to estimate crude odds ratios (OR) and adjusted odds ratios (adj. OR) with 95% confidence intervals (95% CI).

## 3. Results

### 3.1. Demographic Characteristics

Table 1 shows the sociodemographic characteristics of the study population included in the analysis. The prevalence of HTN and DM was 35.1% and 8.6%, respectively, in men and 34.7% and 5%, respectively, in women. The prevalence of HDC was 14.4% in men and 11.8% in women. In men, the mean age was 58.96 ± 0.17 years in the non-HDC group, 62.43 ± 0.24 years in the HTN group, 60.87 ± 0.37 years in the DM group, and 63.87 ± 0.33 years in the HDC group; in women, the mean age was 58.48 ± 0.15 years in the non-HDC group, 65.15 ± 0.21 years in the HTN group, 62.43 ± 0.49 years in the DM group, and 68.24 ± 0.35 years in the HDC group. Except for residential area and SBP, all variables were highly different between men and women. In men, most of the variables had moderate or strong association between non-HDC group and each disease group. Conversely, residential area, alcohol consumption, and dominant hand variables were not associated with HTN, DM, or HDC. In women, all variables were associated with HTN, DM, and HDC, except for smoking status and dominant hand variables. Interestingly, residential area and alcohol consumption were associated with HTN, DM, and HDC only in women and not in men.

### 3.2. Associations of Hypertension, Diabetes, and Comorbidities with Anthropometric Indices and HGS Indices in Men

Table 2, Table 3 and Table 4 present the associations of each disease with anthropometric indices and HGS indices in men. With respect to the associations of HTN with anthropometric and HGS indices, all anthropometric indices had stronger associations with HTN than all absolute and relative HGS indices in all of the models. The anthropometric index BMI showed the strongest association with HTN in Model 1 (adj. OR = 1.63 [1.52–1.76], adj. *p* < 0.001) and Model 2 (adj. OR = 1.75 [1.61–1.90], adj. *p* < 0.001). In terms of the associations of DM with anthropometric indices and HGS, the anthropometric index WC showed the strongest associations in Model 2 (adj. OR = 1.52 [1.34–1.72], adj. *p* < 0.001). Additionally, the anthropometric index WHtR had a stronger association with HDC than with HGS in all models (OR = 2.47 [2.22–2.76], *p* < 0.001 in crude; adj. OR = 2.31 [2.08–2.57], adj. *p* < 0.001 in Model 1; adj. OR = 2.31 [2.08–2.57], adj. *p* < 0.001 in Model 2). Compared with the absolute HGS indices, the relative HGS indices were more strongly associated with HTN, DM, and HDC.

### 3.3. Associations of Hypertension, Diabetes, and Comorbidities with Anthropometric Indices and HGS Indices in Women

Table 5, Table 6 and Table 7 show the associations of each disease with anthropometric indices and HGS indices in women. In HTN, the strongest association was detected for BMI and WHtR in Model 1 (adj. OR = 1.65 [1.55–1.76], adj. *p* < 0.001; adj. OR = 1.66 [1.55–1.77], adj. *p* < 0.001) and in Model 2 (adj. OR = 1.62 [1.52–1.73], adj. *p* < 0.001; adj. OR = 1.61 [1.50–1.73], adj. *p* < 0.001). In DM patients, anthropometric indices such as WC and WHtR had the strongest associations with DM in all models. Also, WHtR showed the strongest association with HDC in all models (OR = 3.20 [2.88–3.56], *p* < 0.001 in the crude model; adj. OR = 2.48 (2.22–2.77), adj. *p* < 0.001 in Model 1; adj. OR = 2.33 (2.06–2.65), adj. *p* < 0.001 in Model 2). Compared to the absolute and relative HGS indices, the relative HGS indices were more strongly associated with HTN, DM, and HDC.

## 4. Discussion

In this study, we investigated the association of HGS and anthropometric indices with HTN, DM, and HDC. Our results revealed that anthropometric indices such as BM, WC, and WHtR are more strongly associated with these diseases than the absolute HGS index or relative HGS indices in both men and women.

To date, many studies have suggested that HGS is strongly associated with HTN and DM [8,14,15,16,17,18,19,20,21,22,23,24,25,26,27]. These studies reported that subjects with HTN or DM have lower HGS than those without HTN [8,18,19,21,22] or DM [8,14,15,16,17,19,20,23,24,25,26,27]. For example, Mainous et al. [8] examined the associations of HGS with DM and HTN based on data from the National Health and Nutrition Examination Survey 2011–2012 in the US and reported that HGS in individuals with undiagnosed and diagnosed HTN and DM was significantly lower than that in individuals without HTN and DM. Most recently, Vaishya et al. [14] reported associations between HGS and various chronic diseases, including cardiovascular disease, HTN, DM, and mental illness, through a narrative review of the literature and reported that HGS was significantly related to cardiovascular disease and DM as well as many other diseases. They argued that HGS is an emerging indicator of vital signs in clinical practice. Ntuk et al. [15] assessed ethnic differences in the association between HGS and the prevalence of DM based on 418,656 white Europeans and South Asian subjects from the UK Biobank. They found that decreased HGS is related to a disproportionately large number of DM cases in black men and South Asian women and men. Shah et al. [19] investigated the relationships of HGS with HTN and DM in older adults in Malaysia and reported that HGS was significantly lower in men with only DM and women with only HTN. Furthermore, Hu et al. [16] evaluated whether HGS is associated with prediabetes in a large population in China and suggested that high HGS is independently related to a decreased prevalence of prediabetes and that HGS is a useful indicator for screening for prediabetes. Cetinus et al. [17] compared HGS and pinching power between a DM group and a control group in Turkey and demonstrated that these metrics were lower in the DM group than in the age-matched control group. In addition, Li et al. [20] examined the association of HGS and skeletal muscle mass with incident DM in a large prospective study of community-dwelling individuals in Australia and reported that low HGS is a risk factor for incident DM in men but that low muscle mass is not associated with incident DM. In Korea, Chon et al. [18] assessed associations of absolute and relative HGS with HTN in a Korean population aged 65 years and older. They argued that absolute and relative HGS are significantly related to HTN, with the relative HGS (HGS/BMI) index having a stronger association with HTN than the absolute HGS index. The relationship between the prevalence of DM and HGS according to the BMI range in Korean adults was examined by Kim et al. [28], who reported that a higher relative HGS (HGS/weight) was associated with a lower prevalence of DM, independent of BMI. Similarly, Kim et al. [21] examined the associations of the prevalence of HTN with relative HGS (HGS/weight) and BMI in Korean elderly subjects based on data from the Korean National Fitness Assessment in 2019 and found that men with low HGS were more likely to have HTN. Our findings are consistent with the results of previous studies suggesting that low HGS is associated with high risk for HTN [8,18,19,21,22] and DM [8,14,15,16,17,19,20,25,26,27]. Additionally, our findings agree with previous results showing that relative HGS is more strongly associated with HTM [18] and DM [28] than is absolute HGS. Several previous studies have suggested that relative HGS is more strongly associated with these diseases than is absolute HGS [14,28]. Relative HGS, which is calculated as the absolute HGS divided by the anthropometric value, can be considered to reflect the body height, weight, and WC of the subject [14]. Therefore, relative HGS indices may help to explain differences in HGS among subjects according to different anthropometry or obesity.

The exact biological and pathophysiological mechanisms linking HGS and HTN or DM are still unknown [8]. However, there are a few potential mechanisms underlying the relationship between HGS and the diseases. In fact, HTN and DM are well known to coexist closely [6,7]. First, one potential mechanism linking HGS and HTN, DM, and HDC may be insulin resistance [6,11,45]. Insulin resistance is a common and essential feature of the pathological mechanism of both DM and HTN [46,47]. HTN is closely linked to insulin resistance [46,48,49,50], and insulin resistance occurs in cardiovascular tissue, skeletal muscle tissue, and adipose tissue and reduces muscle strength [45,46,48,49,50]. Additionally, insulin resistance leads to hypertension via several pathological mechanisms, such as tissue angiotensin II, sympathetic nervous system activity, and inflammatory marker and oxidative stress [48]. Specifically, angiotensin II reduces the metabolic actions of insulin in skeletal muscle [49], and insulin resistance may directly induce high blood pressure by enhancing sympathetic nervous system activity [48,51]. Furthermore, changes in muscle strength or skeletal muscle composition with age lead to low muscle quality and induce metabolic abnormalities such as high blood pressure and insulin resistance [8,52]. DM also accelerates the loss of muscle mass and strength (sarcopenia) due to insulin resistance and glucose toxicity [53,54]. Low HGS is closely related to diabetes and insulin resistance [55], and the positive effects of high HGS may include decreases in weight, adiposity, insulin resistance, and inflammation [56]. Elevated levels of chronic inflammatory markers, such as serum interleukin (IL)-6, C-reactive protein (CRP), and tumor necrosis factor-alpha (TNF-α), are associated with lower HGS and physical function [45,47,55,57,58,59] and with the development of DM and insulin resistance [60,61]. When insulin resistance increases, the production of insulin by pancreatic β-cells also increases. However, this condition does not adapt, and diabetes subsequently develops [62]. Additionally, insulin resistance promotes a decrease in muscle mass and strength [53,54,61]. Although many studies have reported several potential mechanisms for these associations, further research is required to investigate the exact links and mechanisms between DM/HTN and HGS.

This study had several limitations. First, our findings did not establish a cause-and-effect relationship due to the cross-sectional design of the present study. Second, anthropometric indices such as BMI, WC, waist-hip ratio, and WHtR are generally used to measure obesity and obesity-related diseases, and HGS is used to measure muscle strength. We examined two types of indices with different purposes because the anthropometric indices and HGS and combinations of the two types, such as HGS/BMI or HGS/weight, were recently reported as indices to screen for HTN and DM in health-related measurements in large-scale studies and surveys [18,21,28]. Even though the HGS values of normal individuals and individuals with diseases are similar, their weight, WC, and BMI may be different, and high weight, WC, and BMI are more strongly associated with DM and HTN than are low weight, WC, and BMI [21,28]. Therefore, relative HGS, such as HGS/weight, HGS/BMI, HGS/WC, and HGS/WHtR, may be adjusted for the level of obesity [18,28]. Third, although we used adjusted models including many covariates such as sociodemographic characteristics and lipid profiles, we did not consider genetic and metabolic factors and diet composition in the adjusted models due to insufficient or no data. Therefore, further study is needed to examine models that include more covariates expected to influence the diseases. Also, further study is needed to determine whether increasing HGS or muscle strength reduces the risk of HTN, DM, and its comorbidity and to examine whether reducing abdominal obesity or obesity reduces the risk of these diseases, based on longitudinal or interventional studies. Despite these limitations, this study has strengths; the results of this study are statistically powerful because it used large amounts of high-quality data from a nationally representative sample of the South Korean population. Finally, our findings suggest that while it is important to enhance the grip strength or muscle strength for reducing the risks of hypertension, diabetes, and its comorbidity, it is even more important to prevent abdominal obesity. Therefore, we recommend that our results will be used as a guide for local health policies to reduce the risks of these chronic diseases in older people with limited access to local hospitals or public health centers.

## 5. Conclusions

HTN and DM are the most important risk factors for heart disease, stroke, cardiovascular disease and morbidity. In this study, we investigated the association of HGS and anthropometric indices with HTN, DM, and HDC in a Korean population. Although many studies have reported the strong association of HGS with HTN and DM, our findings indicated that anthropometric indices are more strongly associated with HTN, DM, and HDC than the absolute and relative HGS indices in both men and women. To our knowledge, this is the first study to examine HTN, DM, and HDC with anthropometry and HGS indices.

## Figures and Tables

**Table 1:** Demographic characteristics of the subjects.

Variables	Men							Women						
	Non-HDC	HTN	*p* Value	DM	*p* Value	HDC	*p* Value	Non-HDC	HTN	*p* Value	DM	*p* Value	HDC	*p* Value
Numbers (*n*)	2901	2434		596		999		4083	2917		420		993	
Age (years) ***	58.96 ± 0.17	62.43 ± 0.24	<0.001	60.87 ± 0.37	<0.001	63.87 ± 0.33	<0.001	58.48 ± 0.15	65.15 ± 0.21	<0.001	62.43 ± 0.49	<0.001	68.24 ± 0.35	<0.001
Residential area			0.286		0.489		0.094			<0.001		0.009		0.004
Urban	81.36 (1.33)	80.05 (1.55)		80.00 (2.30)		78.26 (2.11)		83.59 (1.21)	79.13 (1.52)		78.02 (2.64)		79.10 (1.90)	
Rural	18.64 (1.33)	19.95 (1.55)		20.00 (2.30)		21.74 (2.11)		16.41 (1.21)	20.87 (1.52)		21.98 (2.64)		20.90 (1.90)	
Education level ***			<0.001		0.029		<0.001			<0.001		<0.001		<0.001
≤Elementary school	16.45 (0.84)	23.01 (1.03)		19.15 (1.68)		25.67 (1.59)		24.07 (0.80)	50.75 (1.18)		40.78 (2.83)		62.50 (1.82)	
Middle school	14.17 (0.77)	16.42 (0.90)		16.79 (1.86)		18.76 (1.38)		17.01 (0.69)	15.50 (0.82)		22.56 (2.41)		14.01 (1.21)	
High school	33.22 (1.03)	31.80 (1.16)		35.23 (2.44)		34.47 (1.77)		38.03 (0.93)	23.66 (1.02)		28.4 (2.64)		16.33 (1.43)	
≥University	36.15 (1.22)	28.77 (1.23)		28.84 (2.29)		21.10 (1.54)		20.89 (0.86)	10.09 (0.68)		8.27 (1.54)		7.16 (0.97)	
Employment status ***			<0.001		<0.001		<0.001			<0.001		0.001		<0.001
No	21.89 (0.85)	31.71 (1.13)		29.62 (2.10)		36.72 (1.75)		47.02 (0.98)	57.16 (1.19)		56.17 (2.75)		67.40 (1.80)	
Yes	78.11 (0.85)	68.29 (1.13)		70.38 (2.10)		63.28 (1.75)		52.98 (0.98)	42.84 (1.19)		43.83 (2.75)		32.60 (1.80)	
Household income ***			0.003		<0.001		<0.001			<0.001		<0.001		<0.001
Low	13.05 (0.70)	20.62 (1.02)		23.52 (2.07)		26.55 (1.64)		16.66 (0.70)	35.03 (1.08)		24.89 (2.29)		43.32 (1.99)	
Middle–low	23.44 (0.97)	25.51 (1.04)		25.13 (1.96)		28.05 (1.65)		24.59 (0.79)	25.40 (0.96)		28.56 (2.45)		25.87 (1.58)	
Middle–high	27.52 (1.04)	23.70 (1.08)		24.85 (2.21)		21.99 (1.52)		25.16 (0.80)	21.30 (0.93)		23.66 (2.47)		18.22 (1.47)	
High	35.98 (1.18)	30.17 (1.24)		26.51 (2.23)		23.41 (1.70)		33.60 (0.99)	18.27 (0.88)		22.89 (2.42)		12.59 (1.37)	
Alcohol consumption ***			0.131		0.127		0.578			<0.001		<0.001		<0.001
No	20.71 (0.91)	18.73 (0.95)		23.92 (2.03)		21.68 (1.51)		39.44 (0.89)	49.10 (1.10)		54.24 (2.88)		57.07 (1.77)	
Yes	79.29 (0.91)	81.27 (0.95)		76.08 (2.03)		78.32 (1.51)		60.56 (0.89)	50.90 (1.10)		45.76 (2.88)		42.93 (1.77)	
Smoking status ***			0.027		0.224		0.471			0.608		0.286		0.320
Everyday	31.63 (1.03)	27.93 (1.15)		33.52 (2.35)		30.79 (1.87)		3.45 (0.36)	3.04 (0.41)		4.31 (1.26)		2.61 (0.53)	
Past	49.55 (1.12)	53.66 (1.22)		51.21 (2.39)		52.05 (2.06)		3.15 (0.31)	3.48 (0.39)		1.88 (0.58)		3.76 (0.63)	
Never	18.81 (0.84)	18.41 (0.97)		15.26 (1.80)		17.16 (1.37)		93.41 (0.48)	93.48 (0.54)		93.81 (1.39)		93.63 (0.86)	
Menopause										<0.001		<0.001		<0.001
No								17.46 (0.75)	6.21 (0.58)		6.28 (1.58)		3.89 (0.86)	
Yes								82.54 (0.75)	93.79 (0.58)		93.72 (1.58)		96.11 (0.86)	
Blood pressure														
SBP (mmHg)	116.6 ± 0.24	132.5 ± 0.38	<0.001	117.5 ± 0.53	0.119	131.0 ± 0.66	<0.001	114.3 ± 0.23	135.4 ± 0.39	<0.001	118.2 ± 0.66	<0.001	133.6 ± 0.71	<0.001
DBP (mmHg) ***	76.10 ± 0.16	82.01 ± 0.30	<0.001	74.06 ± 0.36	<0.001	76.90 ± 0.44	0.082	73.19 ± 0.14	79.54 ± 0.25	<0.001	71.64 ± 0.42	<0.001	74.08 ± 0.41	0.038
Blood parameters														
Glucose (mg/dL) ***	97.68 ± 0.21	100.7 ± 0.24	<0.001	144.7 ± 1.76	<0.001	141.3 ± 1.39	<0.001	94.65 ± 0.16	98.15 ± 0.20	<0.001	135.4 ± 1.67	<0.001	136.7 ± 1.41	<0.001
Glycated hemoglobin (%) **	5.58 ± 0.01	5.66 ± 0.01	<0.001	7.29 ± 0.07	<0.001	7.14 ± 0.05	<0.001	5.61 ± 0.01	5.73 ± 0.01	<0.001	7.14 ± 0.06	<0.001	7.14 ± 0.05	<0.001
Total cholesterol (mg/dL) ***	195.7 ± 0.76	188.7 ± 0.89	<0.001	180.4 ± 1.97	<0.001	173.4 ± 1.64	<0.001	207.2 ± 0.67	197.3 ± 0.84	<0.001	187.8 ± 2.34	<0.001	176.8 ± 1.46	<0.001
HDL cholesterol (mg/dL) ***	47.45 ± 0.24	47.74 ± 0.27	0.431	45.35 ± 0.49	<0.001	44.73 ± 0.45	<0.001	54.70 ± 0.22	52.01 ± 0.28	<0.001	48.84 ± 0.64	<0.001	47.67 ± 0.40	<0.001
Triglycerides (mg/dL) ***	151.6 ± 2.92	165.7 ± 3.35	0.001	177.8 ± 6.60	<0.001	180.9 ± 5.41	<0.001	117.9 ± 1.37	133.2 ± 1.88	<0.001	157.9 ± 6.35	<0.001	151.3 ± 3.75	<0.001
Anthropometrics														
Height (cm) ***	168.8 ± 0.13	167.7 ± 0.15	<0.001	168.4 ± 0.29	0.232	167.9 ± 0.23	<0.001	156.2 ± 0.10	153.9 ± 0.13	<0.001	155.3 ± 0.32	0.009	153.2 ± 0.22	<0.001
Weight (kg) ***	67.62 ± 0.21	69.53 ± 0.25	<0.001	68.96 ± 0.50	0.011	71.60 ± 0.40	<0.001	56.91 ± 0.14	58.81 ± 0.20	<0.001	58.74 ± 0.47	<0.001	60.37 ± 0.35	<0.001
BMI (kg/m^2^) **	23.68 ± 0.06	24.69 ± 0.07	<0.001	24.27 ± 0.15	<0.001	25.35 ± 0.12	<0.001	23.32 ± 0.06	24.8 ± 0.08	<0.001	24.33 ± 0.16	<0.001	25.70 ± 0.13	<0.001
WC (cm) ***	85.04 ± 0.17	88.07 ± 0.20	<0.001	87.76 ± 0.40	<0.001	91.19 ± 0.32	<0.001	79.19 ± 0.16	83.85 ± 0.21	<0.001	83.9 ± 0.46	<0.001	87.76 ± 0.34	<0.001
WHtR ***	0.50 ± 0.00	0.53 ± 0.00	<0.001	0.52 ± 0.00	<0.001	0.54 ± 0.00	<0.001	0.51 ± 0.00	0.55 ± 0.00	<0.001	0.54 ± 0.00	<0.001	0.57 ± 0.00	<0.001
Dominant Hand			0.852		0.305		0.999			0.751		0.658		0.371
Right ***	87.75 (0.70)	88.29 (0.84)		88.70 (1.49)		87.68 (1.24)		89.64 (0.57)	89.4 (0.66)		89.04 (1.82)		88.13 (1.24)	
Left ***	4.96 (0.45)	4.86 (0.57)		5.81 (1.05)		5.00 (0.85)		4.23 (0.38)	4.28 (0.42)		3.65 (1.08)		5.44 (0.82)	
Both ***	7.28 (0.57)	6.84 (0.59)		5.49 (1.11)		7.32 (0.98)		6.13 (0.45)	6.30 (0.51)		7.31 (1.53)		6.43 (0.98)	
Handgrip Strength														
HGS-DH ^A^ (kg) ***	37.49 ± 0.16	36.62 ± 0.19	<0.001	35.52 ± 0.33	<0.001	34.68 ± 0.28	<0.001	22.11 ± 0.09	20.84 ± 0.12	<0.001	21.20 ± 0.27	0.001	19.58 ± 0.19	<0.001
HGS-DH/BMI ^R^***	1.59 ± 0.01	1.49 ± 0.01	<0.001	1.47 ± 0.01	<0.001	1.38 ± 0.01	<0.001	0.96 ± 0.00	0.85 ± 0.01	<0.001	0.88 ± 0.01	<0.001	0.77 ± 0.01	<0.001
HGS-DH/WC ^R^***	0.44 ± 0.00	0.42 ± 0.00	<0.001	0.41 ± 0.00	<0.001	0.38 ± 0.00	<0.001	0.28 ± 0.00	0.25 ± 0.00	<0.001	0.26 ± 0.00	<0.001	0.23 ± 0.00	<0.001
HGS-DH/WHtR ^R^***	74.95 ± 0.35	70.28 ± 0.41	<0.001	68.64 ± 0.71	<0.001	64.41 ± 0.61	<0.001	44.14 ± 0.20	38.84 ± 0.26	<0.001	39.74 ± 0.58	<0.001	34.65 ± 0.40	<0.001
HGS-non-DH ^A^ (kg) ***	38.96 ± 0.17	37.87 ± 0.18	<0.001	37.14 ± 0.34	<0.001	36.43 ± 0.28	<0.001	23.50 ± 0.09	22.12 ± 0.12	<0.001	22.53 ± 0.29	0.001	20.69 ± 0.20	<0.001
HGS-non-DH/BMI ^R^***	1.66 ± 0.01	1.55 ± 0.01	<0.001	1.54 ± 0.01	<0.001	1.45 ± 0.01	<0.001	1.02 ± 0.00	0.90 ± 0.01	<0.001	0.94 ± 0.01	<0.001	0.82 ± 0.01	<0.001
HGS-non-DH/WC ^R^***	0.46 ± 0.00	0.43 ± 0.00	<0.001	0.43 ± 0.00	<0.001	0.40 ± 0.00	<0.001	0.30 ± 0.00	0.27 ± 0.00	<0.001	0.27 ± 0.00	<0.001	0.24 ± 0.00	<0.001
HGS-non-DH/WHtR ^R^***	77.92 ± 0.38	72.70 ± 0.40	<0.001	71.73 ± 0.70	<0.001	67.63 ± 0.61	<0.001	46.92 ± 0.22	41.18 ± 0.27	<0.001	42.20 ± 0.60	<0.001	36.65 ± 0.42	<0.001

*: *p* < 0.05, **: *p* < 0.01, ***: *p* < 0.001. These values represent *p* values for sex differences between all men and women. Continuous data are represented as the mean ± standard error (SE). Categorical data are represented as percentages (SEs). ^A^ Absolute HGS index. ^R^ Relative HGS index. *p* values were obtained using Rao-Scott chi-squared tests for categorical variables and a general linear model for continuous variables. Comparisons were made between the HTN group and non-HDC group, between the DM group and non-HDC group, and between the HDC group and non-HDC group for both men and women. Abbreviations. HGS: handgrip strength, DH: dominant hand, non-DH: nondominant hand, HGS-DH: maximum handgrip strength of the dominant hand, HGS-non-DH: maximum handgrip strength in the nondominant hand, HTN: hypertension, DM: diabetes, HDC: hypertension and diabetes comorbidity, SBP: systolic blood pressure, DBP: diastolic blood pressure, HDL: high-density lipoprotein cholesterol, HT: height, WT: weight, BMI: body mass index, WC: waist circumference, WHtR: waist-to-height ratio, OR: odds ratio, CI: confidence interval.

**Table 2:** Associations of hypertension with anthropometric indices and HGS indices among men.

Variables	Crude		Model 1		Model 2	
	OR (95% CI)	*p* Value	Adj. OR (95% CI)	Adj. *p* Value	Adj. OR (95% CI)	Adj. *p* Value
Anthropometrics						
BMI	1.47 (1.37–1.58)	<0.001	1.63 (1.52–1.76)	<0.001	1.75 (1.61–1.90)	<0.001
WC	1.51 (1.40–1.62)	<0.001	1.55 (1.44–1.67)	<0.001	1.62 (1.50–1.76)	<0.001
WHtR	1.67 (1.55–1.80)	<0.001	1.62 (1.50–1.74)	<0.001	1.69 (1.56–1.84)	<0.001
Handgrip Strength						
HGS-DH ^A^	0.89 (0.83–0.95)	<0.001	1.12 (1.03–1.21)	0.005	1.13 (1.04–1.22)	0.003
HGS-DH/BMI ^R^	0.72 (0.67–0.77)	<0.001	0.81 (0.75–0.88)	<0.001	0.80 (0.74–0.87)	<0.001
HGS-DH/WC ^R^	0.75 (0.70–0.80)	<0.001	0.88 (0.81–0.95)	0.002	0.88 (0.81–0.95)	0.002
HGS-DH/WHtR ^R^	0.74 (0.69–0.79)	<0.001	0.87 (0.80–0.94)	0.001	0.87 (0.80–0.95)	0.001
HGS-non-DH ^A^	0.86 (0.81–0.92)	<0.001	1.09 (1.01–1.17)	0.029	1.11 (1.02–1.20)	0.010
HGS-non-DH/BMI ^R^	0.70 (0.66–0.75)	<0.001	0.80 (0.74–0.86)	<0.001	0.79 (0.73–0.85)	<0.001
HGS-non-DH/WC ^R^	0.73 (0.68–0.78)	<0.001	0.85 (0.79–0.92)	<0.001	0.86 (0.79–0.93)	<0.001
HGS-non-DH/WHtR ^R^	0.72 (0.68–0.77)	<0.001	0.85 (0.79–0.91)	<0.001	0.85 (0.79–0.92)	<0.001

OR and *p* values were obtained from the crude and adjusted analyses using complex sample binary logistic regression. Odds ratios were estimated with 95% confidence intervals. ^A^ Absolute HGS index. ^R^ Relative HGS index. Model 1: Adjusted for age. Model 2: Adjusted for age, residential area, education, employment status, household income, alcohol consumption, smoking status, total cholesterol, HDL cholesterol, and triglycerides. Abbreviations. HGS: handgrip strength, DH: dominant hand, non-DH: nondominant hand, HGS-DH: maximum handgrip strength of the dominant hand, HGS-non-DH: maximum handgrip strength in the nondominant hand, BMI: body mass index, WC: waist circumference, WHtR: waist-to-height ratio, OR: odds ratio, CI: confidence interval.

**Table 3:** Associations of diabetes with anthropometric indices and HGS indices among men.

Variables	Crude		Model 1		Model 2	
	OR (95% CI)	*p* Value	Adj. OR (95% CI)	Adj. *p* Value	Adj. OR (95% CI)	Adj. *p* Value
Anthropometrics						
BMI	1.26 (1.12–1.41)	<0.001	1.33 (1.18–1.49)	<0.001	1.40 (1.23–1.59)	<0.001
WC	1.44 (1.30–1.61)	<0.001	1.47 (1.32–1.63)	<0.001	1.52 (1.34–1.72)	<0.001
WHtR	1.50 (1.34–1.68)	<0.001	1.47 (1.32–1.65)	<0.001	1.51 (1.33–1.71)	<0.001
Handgrip Strength						
HGS-DH ^A^	0.75 (0.67–0.83)	<0.001	0.80 (0.70–0.91)	0.001	0.83 (0.73–0.96)	0.009
HGS-DH/BMI ^R^	0.66 (0.59–0.73)	<0.001	0.69 (0.61–0.77)	<0.001	0.69 (0.61–0.79)	<0.001
HGS-DH/WC ^R^	0.64 (0.58–0.71)	<0.001	0.66 (0.58–0.75)	<0.001	0.68 (0.59–0.78)	<0.001
HGS-DH/WHtR ^R^	0.65 (0.58–0.72)	<0.001	0.67 (0.58–0.76)	<0.001	0.69 (0.59–0.79)	<0.001
HGS-non-DH ^A^	0.78 (0.70–0.86)	<0.001	0.84 (0.74–0.95)	0.004	0.88 (0.77–1.00)	0.051
HGS-non-DH/BMI ^R^	0.68 (0.62–0.75)	<0.001	0.71 (0.64–0.79)	<0.001	0.72 (0.64–0.81)	<0.001
HGS-non-DH/WC ^R^	0.66 (0.60–0.73)	<0.001	0.69 (0.61–0.77)	<0.001	0.70 (0.62–0.80)	<0.001
HGS-non-DH/WHtR ^R^	0.67 (0.61–0.74)	<0.001	0.69 (0.61–0.78)	<0.001	0.71 (0.63–0.82)	<0.001

OR and *p* values were obtained from the crude and adjusted analyses using complex sample binary logistic regression. Odds ratios were estimated with 95% confidence intervals. ^A^ Absolute HGS index. ^R^ Relative HGS index. Model 1: Adjusted for age. Model 2: Adjusted for age, residential area, education, employment status, household income, alcohol consumption, smoking status, total cholesterol, HDL cholesterol, and triglycerides. Abbreviations. HGS: handgrip strength, DH: dominant hand, non-DH: nondominant hand, HGS-DH: maximum handgrip strength of the dominant hand, HGS-non-DH: maximum handgrip strength in the nondominant hand, BMI: body mass index, WC: waist circumference, WHtR: waist-to-height ratio, OR: odds ratio, CI: confidence interval.

**Table 4:** Associations of comorbidities with anthropometric indices and HGS indices among men.

Variables	Crude		Model 1		Model 2	
	OR (95% CI)	*p* Value	Adj. OR (95% CI)	Adj. *p* Value	Adj. OR (95% CI)	Adj. *p* Value
Anthropometrics						
BMI	1.85 (1.68–2.03)	<0.001	2.11 (1.89–2.35)	<0.001	2.11 (1.89–2.35)	<0.001
WC	2.24 (2.03–2.47)	<0.001	2.26 (2.04–2.50)	<0.001	2.26 (2.04–2.50)	<0.001
WHtR	2.47 (2.22–2.76)	<0.001	2.31 (2.08–2.57)	<0.001	2.31 (2.08–2.57)	<0.001
Handgrip Strength						
HGS-DH ^A^	0.67 (0.61–0.73)	<0.001	0.87 (0.78–0.96)	0.008	0.87 (0.78–0.96)	0.143
HGS-DH/BMI ^R^	0.48 (0.43–0.53)	<0.001	0.56 (0.50–0.62)	<0.001	0.56 (0.50–0.62)	<0.001
HGS-DH/WC ^R^	0.48 (0.44–0.54)	<0.001	0.56 (0.50–0.63)	<0.001	0.56 (0.50–0.63)	<0.001
HGS-DH/WHtR ^R^	0.49 (0.44–0.54)	<0.001	0.57 (0.51–0.64)	<0.001	0.57 (0.51–0.64)	<0.001
HGS-non-DH ^A^	0.71 (0.65–0.77)	<0.001	0.94 (0.85–1.05)	0.279	0.94 (0.85–1.05)	0.927
HGS-non-DH/BMI ^R^	0.51 (0.46–0.56)	<0.001	0.60 (0.54–0.66)	<0.001	0.60 (0.54–0.66)	<0.001
HGS-non-DH/WC ^R^	0.51 (0.46–0.56)	<0.001	0.60 (0.54–0.67)	<0.001	0.60 (0.54–0.67)	<0.001
HGS-non-DH/WHtR ^R^	0.51 (0.46–0.57)	<0.001	0.61 (0.55–0.69)	<0.001	0.61 (0.55–0.69)	<0.001

OR and *p* values were obtained from the crude and adjusted analyses using complex sample binary logistic regression. Odds ratios were estimated with 95% confidence intervals. ^A^ Absolute HGS index. ^R^ Relative HGS index. Model 1: Adjusted for age. Model 2: Adjusted for age, residential area, education, employment status, household income, alcohol consumption, smoking status, total cholesterol, HDL cholesterol, and triglycerides. Abbreviations. HGS: handgrip strength, DH: dominant hand, non-DH: nondominant hand, HGS-DH: maximum handgrip strength of the dominant hand, HGS-non-DH: maximum handgrip strength in the nondominant hand, BMI: body mass index, WC: waist circumference, WHtR: waist-to-height ratio, OR: odds ratio, CI: confidence interval.

**Table 5:** Associations of hypertension with anthropometric indices and HGS indices among women.

Variables	Crude		Model 1		Model 2	
	OR (95% CI)	*p* Value	Adj. OR (95% CI)	Adj. *p* Value	Adj. OR (95% CI)	Adj. *p* Value
Anthropometrics						
BMI	1.65 (1.55–1.75)	<0.001	1.65 (1.55–1.76)	<0.001	1.62 (1.52–1.73)	<0.001
WC	1.79 (1.68–1.91)	<0.001	1.59 (1.49–1.70)	<0.001	1.55 (1.45–1.66)	<0.001
WHtR	2.02 (1.89–2.16)	<0.001	1.66 (1.55–1.77)	<0.001	1.61 (1.50–1.73)	<0.001
Handgrip Strength						
HGS-DH ^A^	0.76 (0.72–0.80)	<0.001	1.06 (0.99–1.13)	0.111	1.07 (1.00–1.14)	0.067
HGS-DH/BMI ^R^	0.61 (0.57–0.64)	<0.001	0.79 (0.73–0.84)	<0.001	0.81 (0.76–0.87)	<0.001
HGS-DH/WC ^R^	0.61 (0.57–0.65)	<0.001	0.83 (0.78–0.89)	<0.001	0.86 (0.80–0.93)	<0.001
HGS-DH/WHtR ^R^	0.59 (0.56–0.63)	<0.001	0.83 (0.77–0.89)	<0.001	0.86 (0.80–0.93)	<0.001
HGS-non-DH ^A^	0.75 (0.71–0.79)	<0.001	1.05 (0.98–1.12)	0.147	1.06 (0.99–1.13)	0.101
HGS-non-DH/BMI ^R^	0.59 (0.56–0.63)	<0.001	0.77 (0.72–0.83)	<0.001	0.80 (0.74–0.85)	<0.001
HGS-non-DH/WC ^R^	0.60 (0.57–0.64)	<0.001	0.82 (0.77–0.88)	<0.001	0.85 (0.79–0.91)	<0.001
HGS-non-DH/WHtR ^R^	0.58 (0.55–0.62)	<0.001	0.82 (0.76–0.87)	<0.001	0.85 (0.79–0.91)	<0.001

OR and *p* values were obtained from the crude and adjusted analyses using complex sample binary logistic regression. Odds ratios were estimated with 95% confidence intervals. ^A^ Absolute HGS index. ^R^ Relative HGS index. Model 1: Adjusted for age. Model 2: Adjusted for age, residential area, education, employment status, household income, alcohol consumption, smoking status, total cholesterol, HDL cholesterol, triglycerides, and menopause. Abbreviations. HGS: handgrip strength, DH: dominant hand, non-DH: nondominant hand, HGS-DH: maximum handgrip strength of the dominant hand, HGS-non-DH: maximum handgrip strength in the nondominant hand, BMI: body mass index, WC: waist circumference, WHtR: waist-to-height ratio, OR: odds ratio, CI: confidence interval.

**Table 6:** Associations of diabetes with anthropometric indices and HGS indices among women.

Variables	Crude		Model 1		Model 2	
	OR (95% CI)	*p* Value	Adj. OR (95% CI)	Adj. *p* Value	Adj. OR (95% CI)	Adj. *p* Value
Anthropometrics						
BMI	1.42 (1.28–1.59)	<0.001	1.42 (1.27–1.59)	<0.001	1.32 (1.17–1.49)	<0.001
WC	1.81 (1.61–2.03)	<0.001	1.69 (1.50–1.91)	<0.001	1.56 (1.37–1.77)	<0.001
WHtR	1.88 (1.68–2.10)	<0.001	1.69 (1.49–1.92)	<0.001	1.53 (1.34–1.75)	<0.001
Handgrip Strength						
HGS-DH ^A^	0.81 (0.72–0.92)	0.001	0.97 (0.85–1.11)	0.667	0.98 (0.85–1.12)	0.765
HGS-DH/BMI ^R^	0.69 (0.61–0.79)	<0.001	0.80 (0.70–0.92)	0.001	0.84 (0.73–0.97)	0.020
HGS-DH/WC ^R^	0.64 (0.57–0.73)	<0.001	0.75 (0.65–0.86)	<0.001	0.80 (0.69–0.92)	0.002
HGS-DH/WHtR ^R^	0.64 (0.57–0.72)	<0.001	0.75 (0.66–0.86)	<0.001	0.80 (0.69–0.93)	0.004
HGS-non-DH ^A^	0.81 (0.72–0.92)	0.001	0.98 (0.86–1.11)	0.735	0.99 (0.86–1.12)	0.827
HGS-non-DH/BMI ^R^	0.69 (0.61–0.78)	<0.001	0.80 (0.70–0.91)	0.001	0.84 (0.74–0.96)	0.013
HGS-non-DH/WC ^R^	0.64 (0.57–0.72)	<0.001	0.75 (0.66–0.85)	<0.001	0.79 (0.69–0.91)	0.001
HGS-non-DH/WHtR ^R^	0.64 (0.57–0.72)	<0.001	0.75 (0.66–0.86)	<0.001	0.80 (0.70–0.92)	0.002

OR and *p* values were obtained from the crude and adjusted analyses using complex sample binary logistic regression. Odds ratios were estimated with 95% confidence intervals. ^A^ Absolute HGS index. ^R^ Relative HGS index. Model 1: Adjusted for age. Model 2: Adjusted for age, residential area, education, employment status, household income, alcohol consumption, smoking status, total cholesterol, HDL cholesterol, triglycerides, and menopause. Abbreviations. HGS: handgrip strength, DH: dominant hand, non-DH: nondominant hand, HGS-DH: maximum handgrip strength of the dominant hand, HGS-non-DH: maximum handgrip strength in the nondominant hand, BMI: body mass index, WC: waist circumference, WHtR: waist-to-height ratio, OR: odds ratio, CI: confidence interval.

**Table 7:** Associations of comorbidities with anthropometric indices and HGS indices among women.

Variables	Crude		Model 1		Model 2	
	OR (95% CI)	*p* Value	Adj. OR (95% CI)	Adj. *p* Value	Adj. OR (95% CI)	Adj. *p* Value
Anthropometrics						
BMI	2.10 (1.92–2.29)	<0.001	2.16 (1.96–2.38)	<0.001	2.02 (1.82–2.25)	<0.001
WC	2.75 (2.50–3.03)	<0.001	2.37 (2.14–2.63)	<0.001	2.23 (1.99–2.50)	<0.001
WHtR	3.20 (2.88–3.56)	<0.001	2.48 (2.22–2.77)	<0.001	2.33 (2.06–2.65)	<0.001
Handgrip Strength						
HGS-DH ^A^	0.57 (0.52–0.62)	<0.001	0.90 (0.82–1.00)	0.053	0.93 (0.83–1.03)	0.169
HGS-DH/BMI ^R^	0.40 (0.36–0.44)	<0.001	0.57 (0.51–0.64)	<0.001	0.61 (0.54–0.69)	<0.001
HGS-DH/WC ^R^	0.38 (0.34–0.43)	<0.001	0.58 (0.52–0.65)	<0.001	0.62 (0.55–0.70)	<0.001
HGS-DH/WHtR ^R^	0.37 (0.33–0.41)	<0.001	0.58 (0.51–0.65)	<0.001	0.62 (0.55–0.70)	<0.001
HGS-non-DH ^A^	0.56 (0.51–0.61)	<0.001	0.89 (0.80–0.99)	0.026	0.90 (0.80–1.00)	0.055
HGS-non-DH/BMI ^R^	0.39 (0.35–0.43)	<0.001	0.57 (0.51–0.63)	<0.001	0.59 (0.53–0.67)	<0.001
HGS-non-DH/WC ^R^	0.38 (0.34–0.42)	<0.001	0.58 (0.51–0.65)	<0.001	0.60 (0.53–0.68)	<0.001
HGS-non-DH/WHtR ^R^	0.37 (0.33–0.41)	<0.001	0.57 (0.51–0.64)	<0.001	0.60 (0.53–0.68)	<0.001

OR and *p* values were obtained from the crude and adjusted analyses using complex sample binary logistic regression. Odds ratios were estimated with 95% confidence intervals. ^A^ Absolute HGS index. ^R^ Relative HGS index. Model 1: Adjusted for age. Model 2: Adjusted for age, residential area, education, employment status, household income, alcohol consumption, smoking status, total cholesterol, HDL cholesterol, triglycerides, and menopause. Abbreviations. HGS: handgrip strength, DH: dominant hand, non-DH: nondominant hand, HGS-DH: maximum handgrip strength of the dominant hand, HGS-non-DH: maximum handgrip strength in the nondominant hand, BMI: body mass index, WC: waist circumference, WHtR: waist-to-height ratio, OR: odds ratio, CI: confidence interval.

## Data Availability

The data used in these analyses are available from the Korea National Health and Nutrition Examination Survey (KNHANES) by the Korea Centers for Disease Control and Prevention (KCDC). Anyone can freely access the data (https://knhanes.kdca.go.kr/knhanes/eng/main.do and https://knhanes.kdca.go.kr/knhanes/main.do).
